# Natural intraepithelial lymphocyte populations rise during necrotic enteritis in chickens

**DOI:** 10.3389/fimmu.2024.1354701

**Published:** 2024-02-22

**Authors:** Shuja Majeed, Shaimaa K. Hamad, Bikas R. Shah, Lisa Bielke, Ali Nazmi

**Affiliations:** ^1^ Department of Animal Sciences, College of Food Agriculture and Environmental Sciences, The Ohio State University, Wooster, OH, United States; ^2^ Department of Animal Production, Faculty of Agriculture, Cairo University, Giza, Egypt; ^3^ Prestage Department of Poultry Science, College of Agriculture and Life Sciences, North Carolina State University, Raleigh, NC, United States; ^4^ Food For Health Discovery Theme, The Ohio State University, Columbus, OH, United States

**Keywords:** necrotic enteritis, *Clostridium perfringens*, mucosal immunity, intraepithelial lymphocytes, gene expression, intestine, chickens

## Abstract

Intraepithelial lymphocytes (IEL) reside in the epithelium at the interface between the contents of the intestinal lumen and the sterile environment of the lamina propria. Because of this strategic location, IEL play a crucial role in various immunological processes, ranging from pathogen control to tissue stability. In mice and humans, IEL exhibit high diversity, categorized into induced IEL (conventional CD4 and CD8αβ T cells) and natural IEL (TCRαβCD8αα, TCRγδ, and TCR^neg^ IEL). In chickens, however, the subpopulations of IEL and their functions in enteric diseases remain unclear. Thus, we conducted this study to investigate the role of IEL populations during necrotic enteritis (NE) in chickens. At 14 days of age, sixty-three Specific-pathogen-free (SPF) birds were randomly assigned to three treatments: Control (sham challenge), *Eimeria maxima* challenge (EM), and *Eimeria maxima* + *Clostridium Perfringens* (*C. Perfringens*) co-challenge (EM/CP). The EM and EM/CP birds were infected with *Eimeria maxima* at day 14 of age, and EM/CP birds were additionally orally inoculated with *C. perfringens* at days 18 and 19 of age. Birds were weighed at days 18, 20, and 26 of age to assess body weight gain (BWG). At 20 days of age (1 day-post *C. perfringens* infection; dpi), and 26 days of age (7 dpi), 7 birds per treatment were euthanized, and jejunum was harvested for gross lesion scores, IEL isolation, and gene expression. The EM/CP birds exhibited subclinical NE disease, lower BWG and shorter colon length. The Most changes in the IEL populations were observed at 1 dpi. The EM/CP group showed substantial increases in the total number of natural IEL subsets, including TCRαβ^+^CD4^-^CD8^-^, TCRαβ^+^CD8αα^+^, TCRγδ^+^, TCR^neg^ and innate CD8α (iCD8α) cells by at least two-fold. However, by 7 dpi, only the number of TCRαβ^+^CD4^-^CD8^-^ and TCRαβ^+^CD8αα^+^ IEL maintained their increase in the EM/CP group. The EM/CP group had significantly higher expression of proinflammatory cytokines (IL-1β and IFN-γ) and Osteopontin (OPN) in the jejunum at 1 dpi. These findings suggest that natural IEL with innate and innate-like functions might play a critical role in the host response during subclinical NE, potentially conferring protection against *C. perfringens* infection.

## 1 Introduction

Necrotic Enteritis (NE) presents a significant challenge for the poultry industry, resulting in substantial financial losses of approximately $6 billion annually (1). This disease can manifest in two forms: clinical infection, causing high mortality rates, and subclinical infection, leading to reduced performance (2). Both forms negatively impact the health and profitability of birds in the poultry industry. In the USA, Antibiotic Growth Promoters (AGPs) have been routinely used in poultry operations since 1951 to control NE and other enteric infections (3). However, due to the concerns about antibiotic resistance and the emergence of superbugs, numerous countries, particularly Europe have imposed restriction on AGP usage in poultry (4). These restrictions have resulted in a resurgence of NE and have promoted numerous research efforts to identify feasible alternatives for its control (5–7). Research into antibiotic alternatives has yielded potential strategies for NE control, although none have been as effective as AGPs (6). Therefore, a more comprehensive understanding of various components within the chicken enteric immune system and their interactions with *C. perfringes* is needed. One promising avenue is studying intraepithelial lymphocytes (IEL) response to NE. IEL are located within the intestinal epithelium and represent one of the first lines of defense in encountering enteric infections (8).

Our current knowledge regarding the role of IEL primarily derives from studies conducted in mice and humans, with limited research focused on chickens. These cells have been recognized as an essential arm of mucosal immunity in the intestine, playing a crucial role in maintaining intestinal homeostasis by tolerating food particles and microbiota while eliminating pathogens (9). The IEL populations are composed of diverse subsets of immune cells, with the majority of being T cells (90%) expressing either TCRαβ or TCRγδ receptors (8, 9). These T cells can be further categorized into induced and natural IEL (8). Induced TCR^+^ IEL, including conventional CD4 and CD8αβ T cells, are activated in the periphery upon encountering their specific foreign antigens prior to entering the IEL populations. In contrast, natural IEL (TCRαβ^+^CD8αα^+^ and TCRγδ^+^ subsets) migrate directly to the intestinal epithelium upon their generation (10). The frequencies of T lineage IEL vary across the small intestine and colon in both humans and mice (9–11). In humans, there is a higher frequency of TCRαβ+ IEL compared to TCRγδ+ IEL in the small intestine and colon (12, 13). Conversely, the mouse small intestine has nearly equal proportions of both populations, with a shift toward TCRαβ+ IEL in the colon. In both human and mouse, the colon maintains a higher proportion of TCRαβ^+^CD4^+^ IEL than the small intestine (13). However, the opposite is observed for TCRαβ^+^CD8αβ^+^ IEL. In mice, 20-50% of TCRαβ+ IEL in the intestine can be TCRαβ+CD8αα+, while in humans, this population constitutes less than 1% of TCRαβ+ IEL (11). Finally, approximately 10% of IEL are non-T cells known as TCR^neg^ IELs, which include innate lymphoid-like cells (14–16), intracellular CD3, and iCD8α (17, 18).

In the chickens, the presence of IEL was first reported by Lawn and his colleagues in 1988, using light and electron microscopy (19). Their findings indicated that the majority of chicken IEL are T-lineage lymphocytes. Subsequent later studies, while confirming the majority of IEL are T cells, demonstrated that natural killer (NK) cells also make up a significant portion of IEL (20, 21). Moreover, the role of IEL subsets mediating chicken responses to enteric diseases was limited to CD4^+^, CD8^+^, and TCRγδ^+^ T cells, which increased in frequency and changed their gene expression profiles during *Eimeria* infection (22–24). However, the response of subsets to NE has not been investigated. Therefore, the main aim of this study is to characterize IEL subsets using flow cytometry during *C. perfringens*-induced NE in SPF chickens. We employed a co-infection approach involving *Eimeria maxima* followed by *C. perfringens* to induce NE. *Eimeria* parasites are recognized as the primary predisposing factor for NE due to their ability to disrupt the integrity of the intestinal epithelium (25, 26). This disruption leads to the secretion of mucus and leakage of nutrients into the intestinal lumen, creating an environment that attracts *C. perfringens* to the small intestine and facilitates colonization (27, 28). Our results indicate that the numbers of natural IEL subsets, including TCRαβ^+^CD4^-^CD8^-^, TCRαβ^+^CD8αα^+^, TCRγδ^+^, TCR^neg^, and iCD8α^+^, increased during an early *C. perfringens* infection. Additionally, *C. perfringens* infection induced expression of OPN, IL-1β, and IFN-γ genes at 1 dpi (day-post C. *perfringens* infection).

## 2 Materials and methods

### 2.1 Ethics statement

The animal in the research trial was approved by The Ohio State University’s Institutional Animal Care and Use Committee (IACUC 2022A00000026) and conducted in compliance with the guidelines and regulations.

### 2.2 Husbandry, experimental design, and performance

Sixty-three SPF birds were placed in a single-floor pen with a sawdust litter in an environmentally controlled house (**Supplementary Figure 1**). The birds were divided into three groups (control, EM, and EM/CP) of 21 birds each and placed in three separate pens. On day 14 of age, the control group underwent a mock challenge with phosphate buffer saline (PBS, Quality Biological), while the EM and EM/CP groups were orally challenged with 5x10^3^ sporulated oocysts/ml/bird of *Eimeria maxima* (provided generously by Dr. Lisa Beilke). On days 18 and 19 of age, the EM/CP group was orally inoculated with *C. perfringens* strain type A (CP1 [netB^-^] (29), provided by Dr. Lisa Beilke) at a concentration of 1x10^8^ CFU/ml, while the EM group was administered PBS. Monitoring for signs of illness, mortality, and body weight was carried out starting from day 18 of age and continued throughout the duration of the experiment. Body weight gain (BWG) was calculated from days 18 to 20 and days 18 to 26 of age. At day 20 (1dpi) and 26 (7dpi) of age, 7 birds per group were euthanized through CO_2_ asphyxiation for sample collections.

### 2.3 Lesion scores and intestinal length

At 1 dpi, the jejunum-ileum junction was examined for NE lesions using 0-4 scale system (30), where 0 denotes no gross lesions, 1 signifies a thin-walled or friable intestine, 2 indicates focal necrosis or ulceration, 3 means large patches of necrosis, and 4 represents a severe, extensive necrosis. Additionally, the lengths of the duodenum, jejunum, ileum, and colon were individually measured for each bird.

### 2.4 IEL isolation and flowcytometry

**Figure 1:**
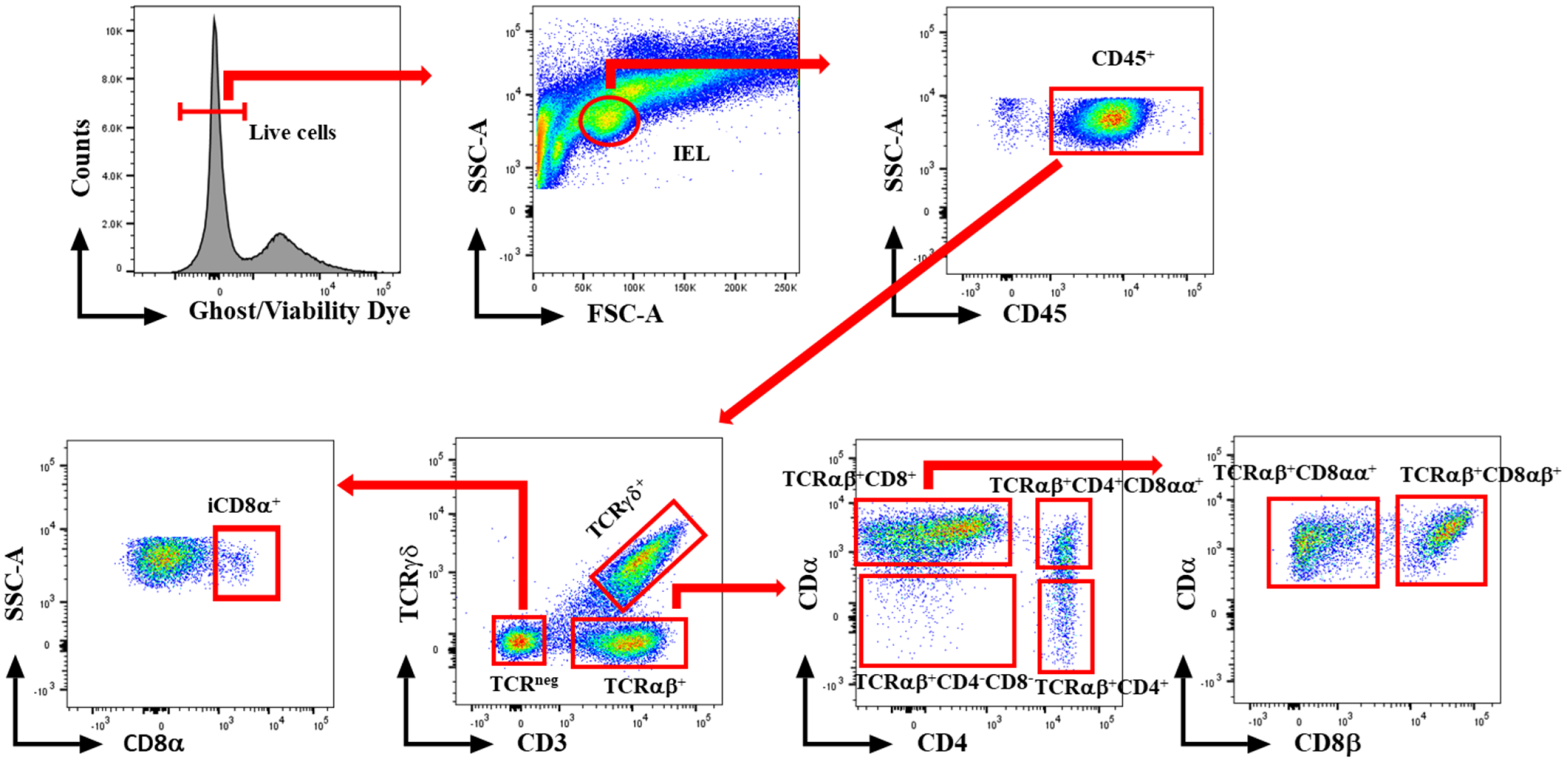
Gating strategy for flowcytometric analysis of IEL populations in jejunum of chickens. Cells were stained with ghost Red 510 and anti-chicken CD45 SPRD, CD4 PE-CY7, CD3 AF547, TCRγδ FITC, CD8α AF700, and CD8β PE antibodies. Gates were created in FlowJo.

At 1 and 7 dpi, approximately 10 cm segment of the jejunum was subjected to mechanical disruption, following a previously established protocol (31). In brief, the intestinal tissues were thoroughly rinsed with PBS and longitudinally opened to remove mucus and fecal matters. The tissue was cut into approximately 1 cm pieces and agitated in PBS supplemented with 5% chicken serum (Sigma-Aldrich, St. Louis, MO, USA), 2 mM EDTA (Quality Biological, Gaithersburg, MD, USA), and 2 mM dithiothreitol (Sigma-Aldrich, USA) at 150 rpm, 37°C for 45 minutes. Subsequently, the supernatant was passed through a gauze column, and the IEL fraction was enriched from the supernatants using a discontinuous 40/70% Percoll density gradient (Cytiva, Marlborough, MA, USA). The recovered cells were incubated for 5 minutes with ACK buffer (Quality Biological, USA) to lyse red blood cells and resuspended in a staining buffer. The number of live cells was counted using the trypan blue exclusion method. Then, the immune cells were stained with fluorochrome-conjugated anti-chicken CD45 SPRD (LT40), CD4 PE-CY7 (CT-4), CD3 AF547 (CT-3), TCRγδ FITC (TCR-1), CD8α AF700 (CT-8), and CD8β PE (EP42) antibodies (Southern Biotech, Birmingham, AL, USA). To differentiate between live and dead cells, we further stained cells with ghost viability dye-Red 510 (Tonbo Biosciences, San Diego, CA, USA). The frequency of stained cells was acquired using a flow cytometer BD FACSCanto II (BD Biosciences, Franklin Lakes, NJ, USA), and data analysis was carried out using FlowJo v10.8.1 software (BD Biosciences, USA). **Figure 1** shows the gating strategy for IEL subsets. Data was reported as the number of cells per gram of tissue.

### 2.5 RNA isolation and gene expression

At 1 and 7dpi, about 1 cm from the distal portion of the jejunum tissues were collected in RNA later and stored in -80° C until further analysis. RNA was isolated from jejunum tissues using the Monarch^®^ Total RNA Miniprep Kit (New England Biolabs^®^, Ipswich, MA, USA) according to the standard protocol. Subsequently, cDNA was synthesized from the RNA using the LunaScript^®^ RT SuperMix Kit (New England Biolabs^®^, USA). Quantitative real-time PCR was then conducted on a Bio-Rad CFX connect machine using Luna^®^ Universal qPCR Master Mix (New England Biolabs^®^, USA) for OPN, IL-1β, IFN-γ, TGF-β, and TNF-α genes. The primer sequences are shown in **Table 1**. The cycle threshold (Ct) for each gene was normalized to the housekeeping gene, GAPDH. Relative fold change was calculated in comparison to the control group using the 2^- ΔΔCt^ method (32).

**Table 1:** List of sequences of primers used for quantitative real-time PCR.

Gene	Accession NO.	Primer sequence (5’-3’)	Amplicon size (bp)
OPN	NM_204535.4	F: AAGAGGCCGTGGATGATGATG	254
		R: ATCCTCAATGAGCTTCCTGGC	
IL-1β	XM_015297469.1	F: CCCGCCTTCCGCTACA	66
		R: CACCARGCACTTCTGGTTGATG	
IFN-γ	NM_205149.1	F: GCTCCCGATGAACGACTTGA	63
		R: TGTAAGATGCTGAAGAGTTCATTCG	
TNF-α	MF000729.1	F: CCCATCCCTGGTCCGTAAC	77
		R: ATACGAAGTAAAGGCCGTCCC	
TGF-β	NM_001318456.1	F: GCCGACACGCAGTACACCAAG	54
		R: GCAGGCACGGACCACCATATTG	
GAPDH	NM_204305	F: CCTAGGATACACAGAGGACCAGGTT	64
		R: GGTGGAGGAATGGCTGTCA	

OPN, osteopontin or Spp-1; IL-1β, interleukin 1beta; IFN-γ, interferon gamma; TNF-α, tumor necrosis factor alpha; TGF-β, tumor growth factor beta; GAPDH, Glyceraldehyde 3-phosphate dehydrogenase; F, forward; R, reverse.

### 2.6 Statistical analysis

Data analysis was performed using GraphPad PRISM v10.0.3 software (GraphPad, Boston, MA, USA). Flow cytometric data and lesion scores underwent analysis through a one-way non-parametric test (Kruskal-Wallis), followed by Dunn’s test. For BWGs, intestinal length, and gene expression, One-way ANOVA was conducted, followed by the Dunnett test. Multiple comparison tests were employed to distinguish means between groups. The results were expressed as mean ± standard error of the mean (SEM), with statistical significance set at *P* < 0.05.

## 3 Results

### 3.1 *Emeria Maxima* and *C. perfringens* co-infection induced mild NE disease

**Figure 2:**
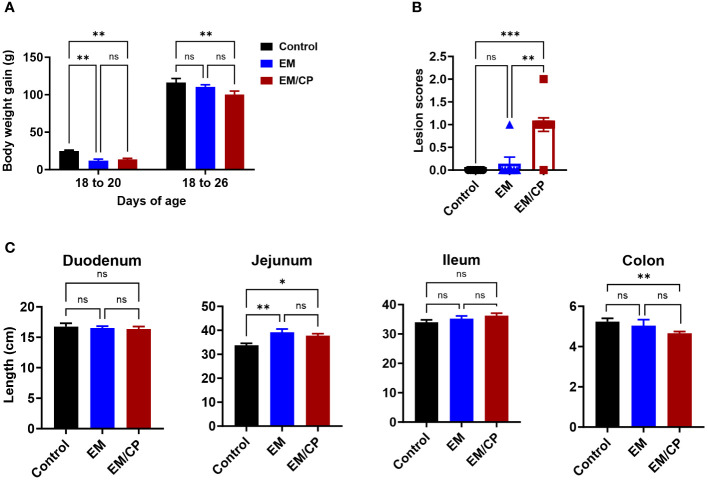
*Eimeria* challenge followed by *C*. *Perfringes* infection induced mild NE disease. **(A)** Body weight gain per gram from day 18-20 and 18-26 of age. One-way ANOVA and Dunnett tests. **(B)** Gross lesion scores of jejunal-ileum junctions at 1 dpi (day 20 of age). Control group received PBS. EM group infected with *Eimeria maxima*; at day 14 of age. EM/CP group infected with Eimeria maxima at day 14 of age and *C*. *perfringens* at days 18 and 19 of age. One-way Kruskal-Wallis and Dunn’s tests. Each dot represents an individual bird. **(C)** Length of intestinal sections in cm at 1 dpi (day 20 of age). One-way ANOVA and Dunnett tests. ns, non-significance, *P<0.05; **P<0.01; ***P<0.001.

Throughout the course of *C. perfringens* infection, no instances of mortality or morbidity were recorded in any of the groups. Body weights were documented for each group on days 18, 20, and 26 of age. Body weight gain (BWG) was calculated for two intervals (18-20 and 18-26). In both intervals, the EM/CP group exhibited significantly lower BWG (*P* < 0.01) compared to the non-infected control group (**Figure 2A**). However, BWG was comparable between the EM/CP and EM groups. Only at 18-20 interval, the EM showed significantly reduced BWG (*P < 0.01*) compared to the control group. At 1 dpi, the EM/CP group displayed the highest lesion scores (score 1) in the jejunum-ileum junction compared to the control and EM groups (**Figure 2B**).

Colon length shortening is commonly associated with intestinal inflammation and is considered a biological marker in various colitis models in mice (33–36). Hence, we investigated whether NE led to alterations in the length of intestinal sections at 1 dpi (**Figure 2C**). The EM/CP and EM groups exhibited a significantly longer jejunum length and a shortened colon length compared to the control group (*P*<0.05, and *P < 0.01*, respectively). However, the jejunum and colon lengths were comparable in the EM/CP and EM groups. Additionally, there were no discernible changes in the lengths of the duodenum and ileum between the groups. These findings suggest that *Emeria Maxima* and *C. perfringens* co-infection induced a mild or subclinical NE disease under our experimental conditions.

### 3.2 Natural IEL increases during the early incidence of NE

**Figure 3:**
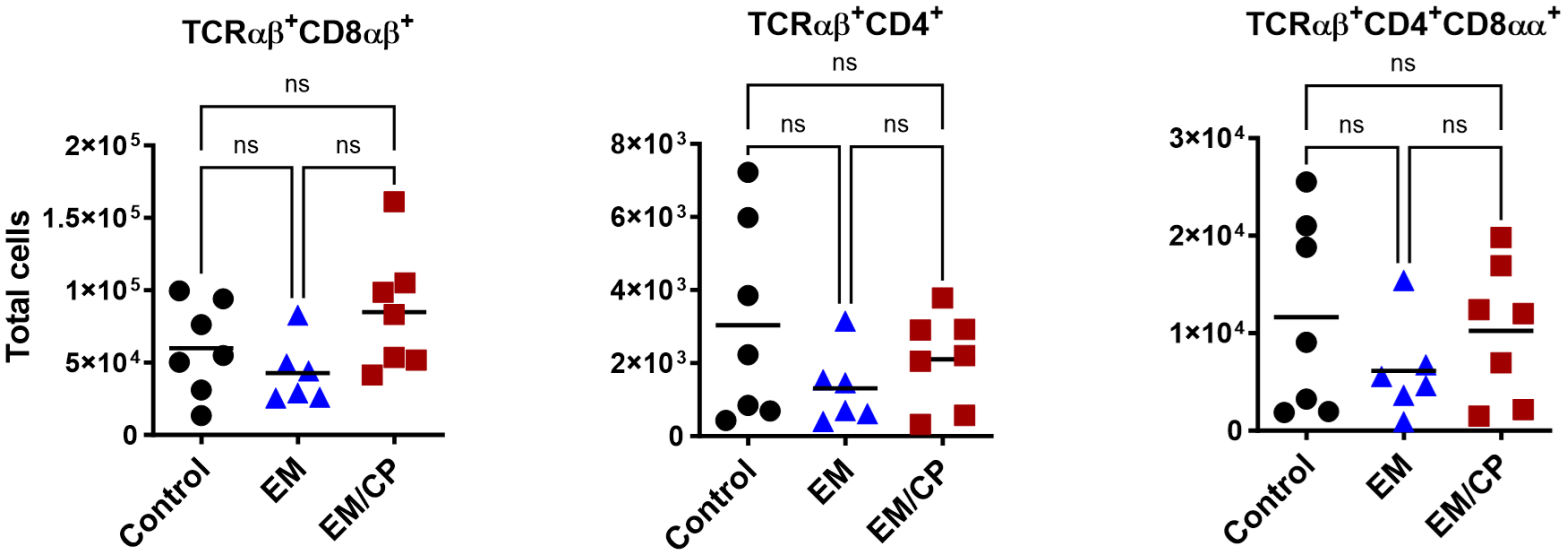
The Number of induced IEL was not impacted by NE disease at 1 dpi. Control group received PBS. EM group infected with *Eimeria maxima*; at day 14 of age. EM/CP group infected with *Eimeria maxima* at day 14 of age and *C. perfringens* at days 18 and 19 of age. TCRαβ^+^ cells are CD45^+^CD3^+^ TCRγδ^-^. Total cell number/gram of jejunum. One-way Kruskal-Wallis and Dunn’s tests. Each dot represents an individual bird. Black bar depicts mean value. ns, non-significance.

**Figure 4:**
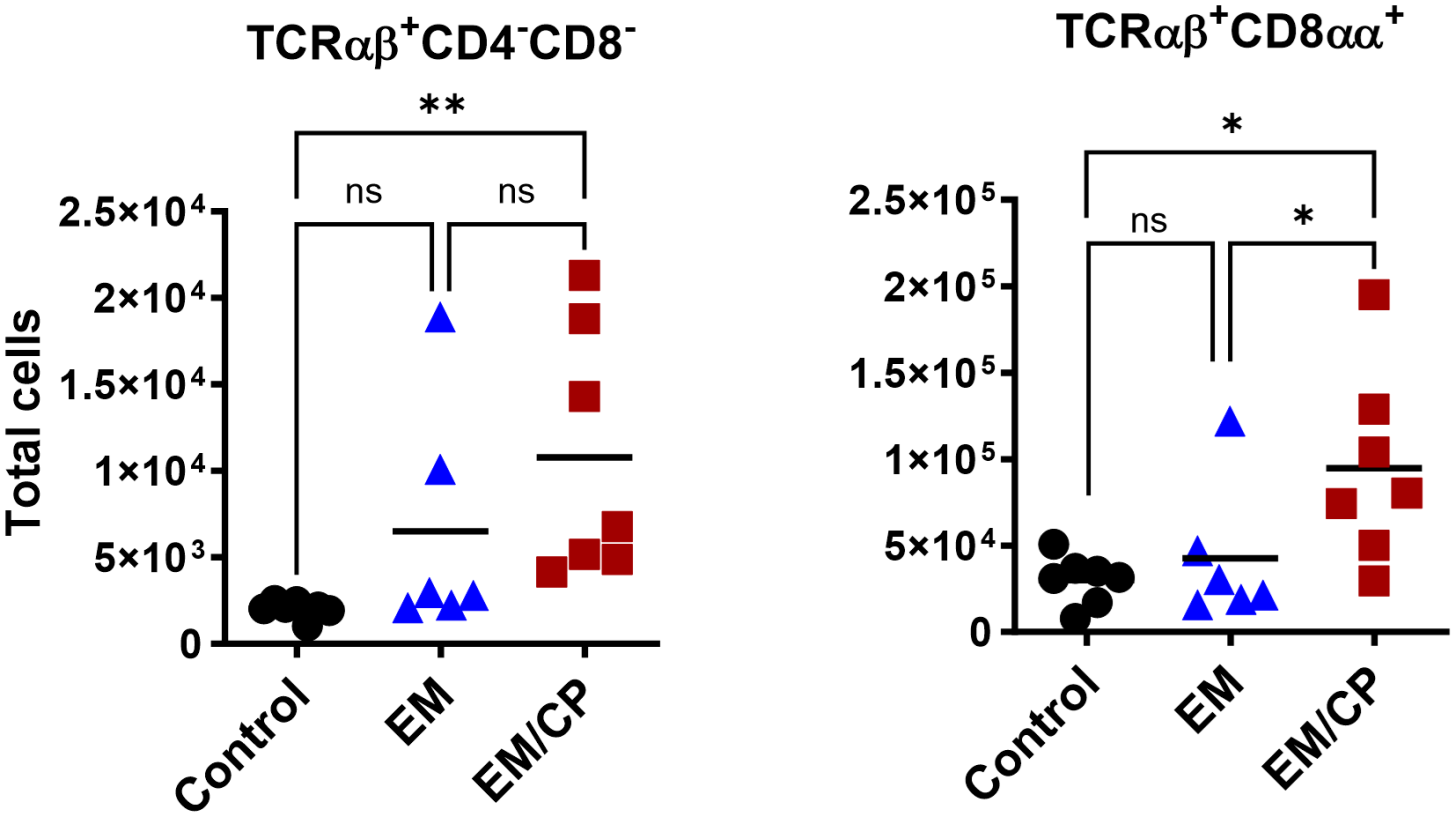
The number of natural TCRαβ^+^CD8αα^+^ IEL increased following NE disease at 1 dpi. Control group received PBS. EM group infected with *Eimeria maxima*; at day 14 of age. EM/CP group infected with *Eimeria maxima* at day 14 of age and *C. perfringens* at days 18 and 19 of age. TCRαβ^+^ cells are CD45^+^CD3^+^ TCRγδ^-^. Total cell number/gram of jejunum. One-way Kruskal-Wallis and Dunn’s tests. Each dot represents an individual bird. Black bar depicts mean value. ns, non-significance; *P<0.05; **P<0.01.

**Figure 5:**
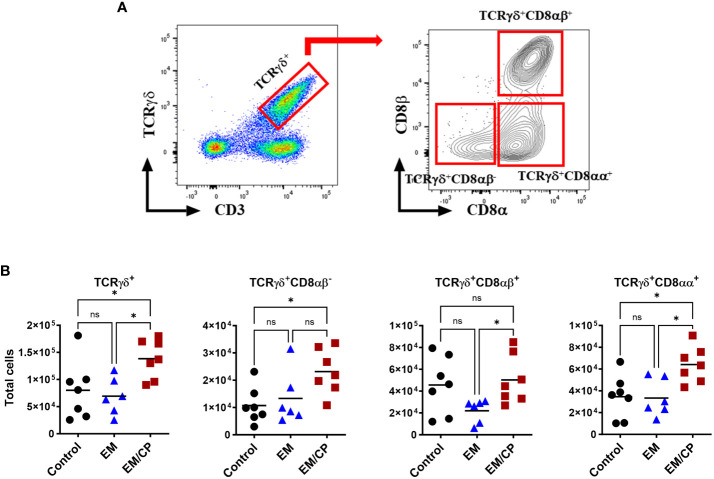
The number of natural TCRγδ IEL subsets increased following NE disease at 1 dpi. **(A)** Gating strategy used to differentiate TCRγδ IEL subpopulations. **(B)** The number of TCRγδ IEL subsets/gram of jejunum. Control group received PBS. EM group infected with *Eimeria maxima*; at day 14 of age. EM/CP group infected with *Eimeria maxima* at day 14 of age and *C. perfringens* at days 18 and 19 of age. TCRγδ^+^ cells are CD45^+^CD3^+^ TCRγδ^+^. One-way Kruskal-Wallis and Dunn’s tests. Each dot represents an individual bird. Black bar depicts mean value. ns, non-significance; *P<0.05.

**Figure 6:**
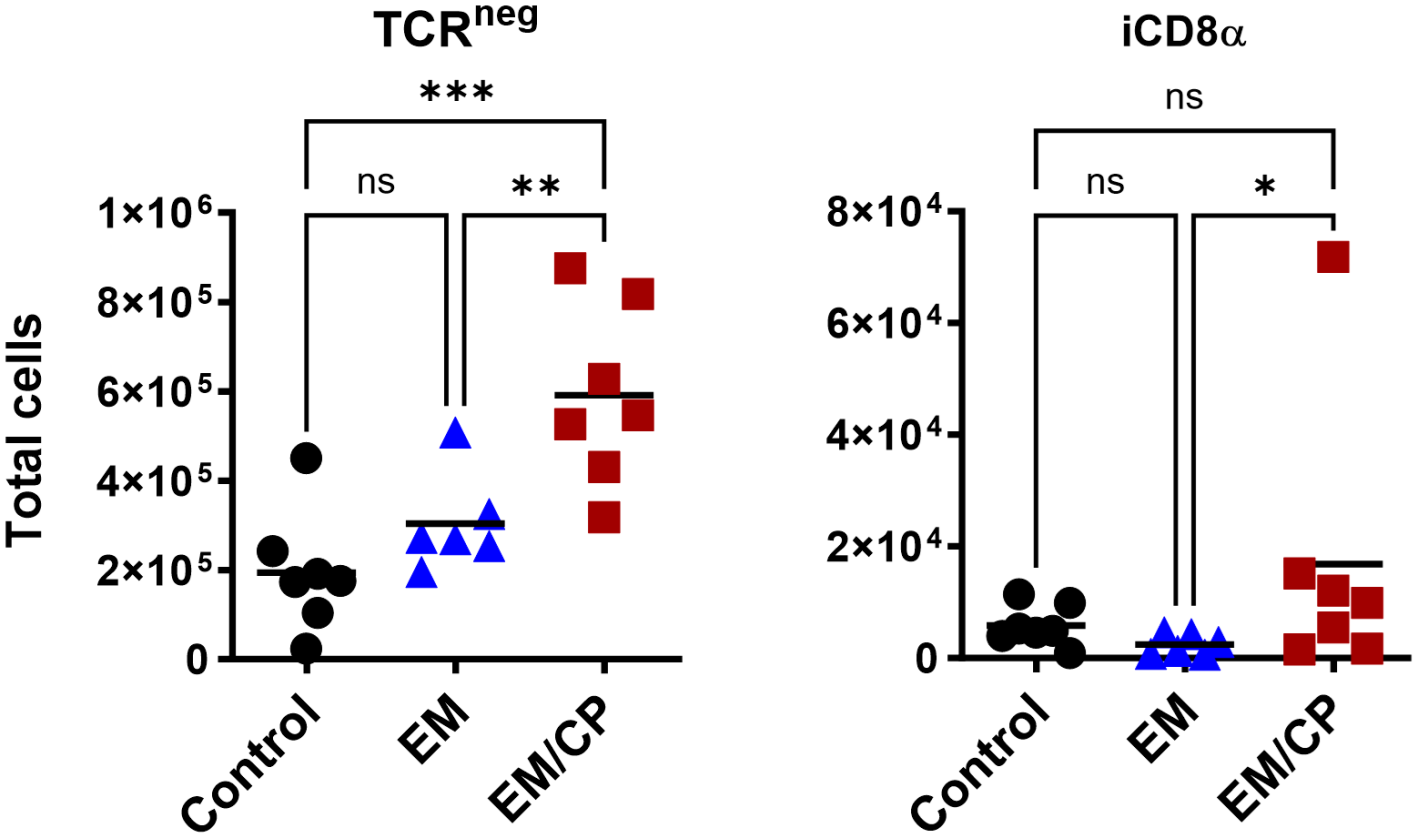
The number of natural TCR^neg^ IEL induced following NE disease at 1 dpi. Control group received PBS. EM group infected with *Eimeria maxima*; at day 14 of age. EM/CP group infected with *Eimeria maxima* at day 14 of age and *C. perfringens* at days 18 and 19 of age. TCR^neg^ cells are CD45^+^CD3^-^. Total cell number in jejunum/gram. One-way Kruskal-Wallis and Dunn’s tests. Each dot represents an individual bird. Black bar depicts mean value. ns, non-significance; *P<0.05; *P<0.05; **P<0.01; ***P<0.001.

To explore the involvement of Intraepithelial Lymphocyte (IEL) populations during NE disease, we examined the IEL subpopulations in jejunum tissues following *C. perfringens* infection at 1 and 7 dpi. **Figure 1** outlines the gating strategy applied for flow cytometric analysis of IEL subsets. At 1 dpi, there were no alterations in the number of induced IEL populations (TCRαβ^+^CD8αβ^+^, TCRαβ^+^CD4^+^, TCRαβ^+^CD4^+^CD8αα^+^) across all groups (**Figure 3**). However, there were notable increases in the number of jejunal natural IEL subpopulations after *C. perfringens* infection at 1 dpi (**Figures 4**–**6**). The EM/CP groups demonstrated over a two-fold increase in the number of TCRαβ^+^CD8αα^+^ cells compared to the control and EM groups (**Figure 4**). The number of TCRαβ^+^ IEL lacking the expression of CD4 and CD8 receptors significantly (*P*<0.01) increased in the EM/CP group compared to the control group only.

In mice, TCRγδ T cells constitute a small fraction of circulating lymphocytes in the blood and peripheral tissues (37), and these cells make up about 40-70% of the total IEL population, with 80% of TCRγδ cells expressing CD8αα homodimers (38). Chickens, on the other hand, have a substantial fraction of TCRγδ T cells in the blood and various tissues, including the intestine (39). In the current study, the total number of TCRγδ cells was significantly (*P*<0.05) increased by twofold in the EM/CP group compared to the other two groups (**Figure 5**). Because intestinal TCRγδ T cells in chickens express CD8αα homodimers or CD8αβ heterodimers (40), we further analyzed the TCRγδ^+^ IEL subpopulations in the jejunum based on their expression of CDαα and/or CDαβ receptors. The EM/CP group had more TCRγδ^+^CD8αα^+^ and TCRγδ^+^CD8αβ^-^ cells compared to the non-infected control group, while their numbers of TCRγδ^+^CD8αα^+^ and TCRγδ^+^CD8αβ^+^ cells were increased compared to the EM group. Then, we examined a fraction of non-T cell IEL that do not express CD3 receptor, TCR^neg^ IEL (**Figure 6**). There was a significant surge in the number of TCR^neg^ IEL in jejunum following *C. perfringens* infection compared to the other groups. Among TCR^neg^ cells, the number of iCD8α^+^ cells increased in the EM/CP group compared to the EM group but not to the control group at 1dpi.

**Figure 7:**
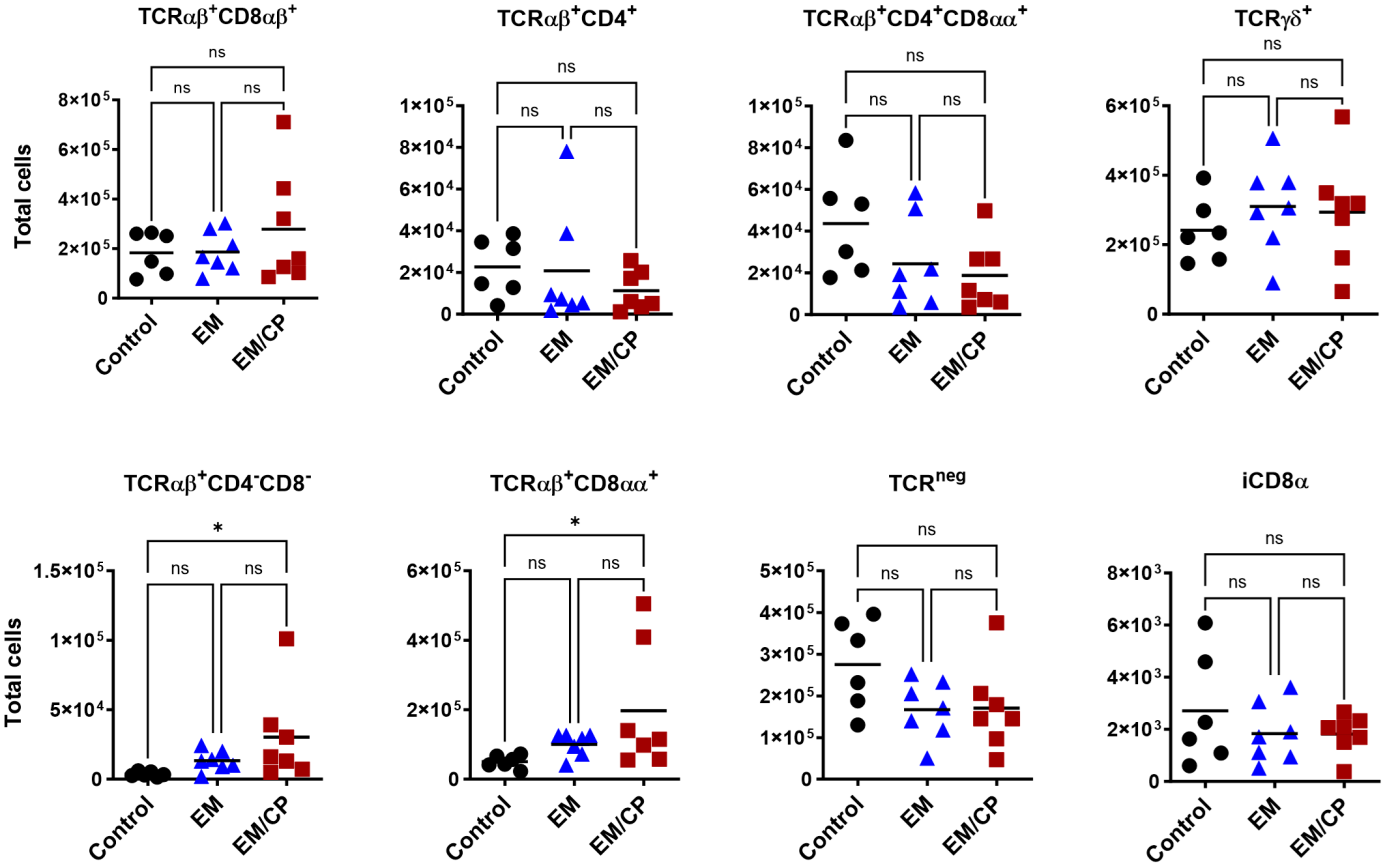
The changes in IEL subsets in the jejunum at 7dpi, were limited to TCRαβ^+^CD4^-^C8^-^ and TCRαβ^+^CD8αα^+^ cells. Control group received PBS. EM group infected with *Eimeria maxima*; at day 14 of age. EM/CP group infected with *Eimeria maxima* at day 14 of age and *C. perfringens* at days 18 and 19 of age. Total cell number/gram of tissue. One-way Kruskal-Wallis and Dunn’s tests. Each dot represents an individual bird. Black bar depicts mean value. ns, non-significance; *P<0.05.

At 7dpi, there were no changes in the number of most IEL subpopulations in the jejunum, except for TCRαβ^+^CD4^-^CD8^-^ and TCRαβ^+^CD8αα^+^ cells (**Figure 7**). The EM/CP group maintained a significant greater number of these cells compared to the control group. These results indicate that natural IEL might play a critical intestinal defense during the early stage of NE.

### 3.3 OPN expression in the jejunum is associated with early *C. perfringens* infection

**Figure 8:**
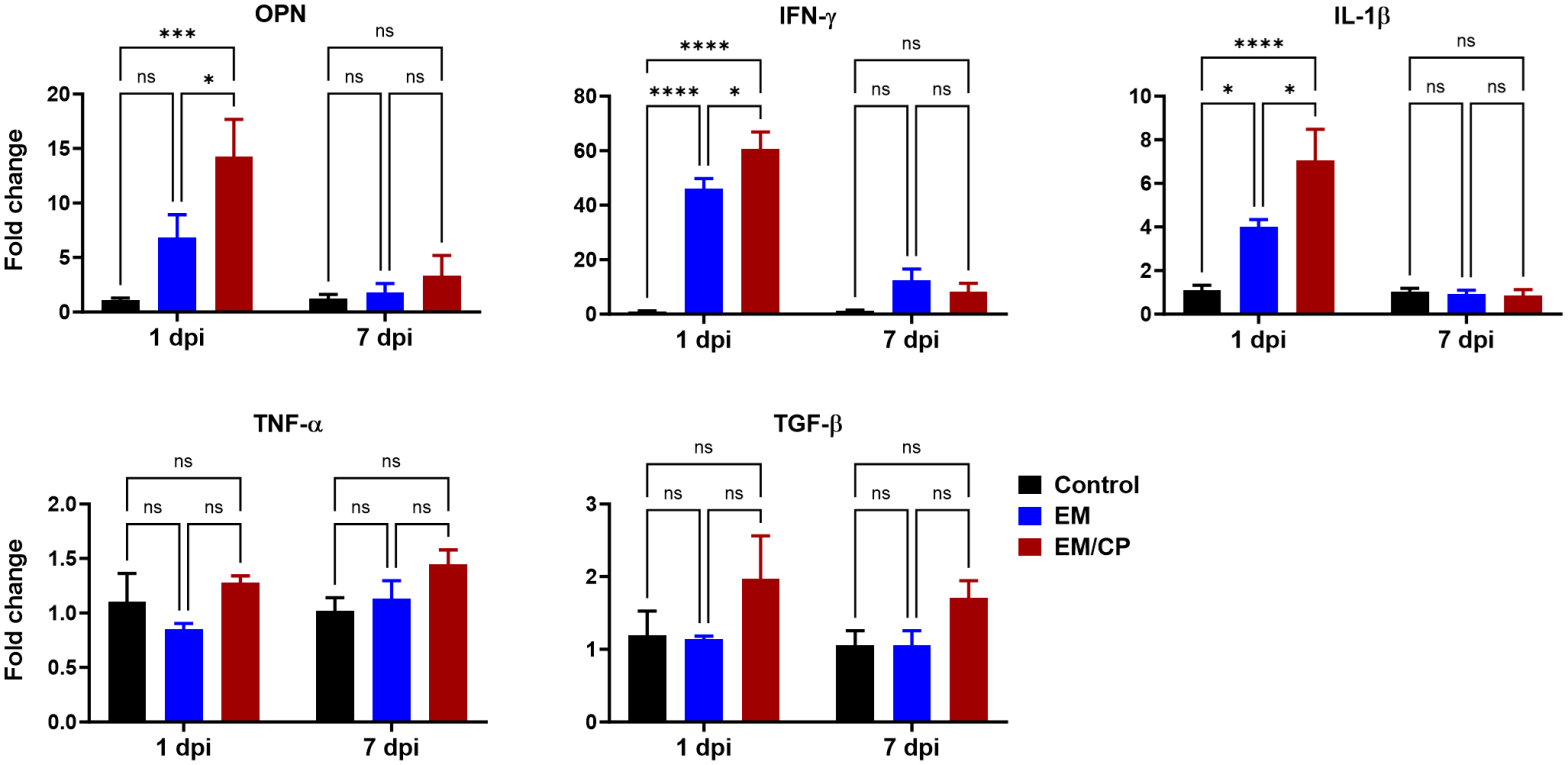
Gene expression of selected genes in jejunum following NE disease at 1dpi and 7dpi. Control group received PBS. EM group infected with *Eimeria maxima*; at day 14 of age. EM/CP group infected with *Eimeria maxima* at day 14 of age and *C. perfringens* at days 18 and 19 of age. OPN, osteopontin or Spp-1; IL-1β, interleukin 1beta; IFN-γ, interferon gamma; TNF-α, tumor necrosis factor alpha; TGF-β, tumor growth factor beta. One-way ANOVA and Dunnett tests. Fold change presented as mean ± SEM. ns, non-significance; *P<0.05; ***P<0.01; ****P<0.001.

OPN is a pleiotropic cytokine encoded by the Spp-1 gene and plays a crucial role in various biological processes. Our previous findings in mice indicate that OPN is primarily secreted by iCD8α cells in the intestine, and together, they serve as important mediators of IEL homeostasis by providing survival and proliferation signals for most IEL populations (31, 41). Observing an increase in the number of iCD8α cells in the jejunum after *C. perfringens* infection at 1 dpi, we investigated whether the expression of the OPN gene is associated with the rise in the number of iCD8α cells. At 1 dpi, the expression of OPN mRNA in the jejunum of the EM/CP group was significantly upregulated by 15 and 2 folds compared to the control (*P*<0.001), and the EM groups (*P*<0.05), respectively (**Figure 8**). There was no change in the expression of OPN between groups at 7 dpi. Additionally, we assessed the expression of different inflammatory and anti-inflammatory cytokines. Similar to the OPN pattern, the expressions of IFN-γ, and IL-1β genes were significantly upregulated in the EM/CP group compared to the control (*P*<0.0001) and EM (*P*<0.05) groups at 1 dpi, but not at 7 dpi (**Figure 8**). In addition, The EM group displayed significant upregulation of IFN-γ and IL-1β genes at 1 dpi only. On the other hand, there were no changes in the expression of TGF-β and TNF-α genes between groups at all time points.

## 4 Discussion

*C. perfringens* is an opportunistic bacterium known to trigger NE outbreaks in broiler chickens, particularly in the presence of predisposing factors such as stress, immunosuppression, an imbalanced diet, and intestinal damage caused by *Eimeria* infection (26). The outcomes of NE pathogenicity can manifest as either clinical infection, characterized by a high mortality rate and severe intestinal damage, or subclinical infection, associated with low mortality, mild intestinal damage, malnutrition, and reduced body weight gain (30, 42). In our experimental conditions, *C. perfringens* infection induced subclinical NE disease, as evidenced by the absence of mortality, reduced BWGs, and mild intestinal damage (average lesion scores = 1; **Figure 2**). Although the EM/CP group exhibited the lowest BWGs, these gains were not statistically significant compared to EM group. This lack of significance could be attributed to factors such as the chicken background (SPF layer chicks in this study), their age, and virulence of *C. perfringens* strain (25). At 14 days of age, birds might be more susceptible to EM infection with 5x10^3^ oocysts, which conceals the differences in BWGs between EM/CP and EM groups (43). The signs of subclinical NE were accompanied with upregulation of proinflammatory cytokine genes IL-1β and IFN-γ, but not TNF-α at 1 dpi, which is necessary for the activation of innate immune cells such as macrophages (**Figure 8**). It is common in murine models of colitis that intestinal inflammation alters colon length (33–36). Our data indicated that *C. perfringens* infection shortened the colon length at 1 dpi (**Figure 2C**), suggesting potential colonic inflammation. Interestingly, mice infected with porcine *C. perfringens* displayed a shortened colon length in an infectious dose-dependent manner (44).

Intestinal IEL which are interspersed at the basolateral side of epithelial cells, represent the primary immunological defense against microbial invaders. To deepen our understanding of host-pathogen interactions during NE disease, we performed a flow cytometric analysis of IEL compartments in the jejunum following *C. perfringens* infection at 1 and 7 dpi. During the early stage of infection at 1 dpi, the EM/CP-infected group exhibited substantial numbers of natural IEL subpopulations, including TCRαβ^+^CD4^-^CD8^-^, TCRαβ^+^CD8αα^+^, TCRγδ^+^, and TCR^neg^ cells in the jejunum compared to the control and EM groups (**Figures 4**–**6**). The first three cell types are subsets of T cells known as “innate-like T cells,” capable of interacting with non-classical MHC molecules and, therefore, do not require antigen presentation for their functions (45, 46). The TCRαβ^+^CD4^-^CD8^-^ cells generated in the thymus, migrate directly to the intestinal epithelium, where they gain expression of CD8αα homodimers to become TCRαβ^+^CD8αα^+^IEL (47–49). TCRαβ^+^CD8αα^+^ cells are exclusively part of the IEL compartment, constituting 20-50% and less than 1% of T-cell IEL in mice and humans, respectively (11, 50). Our current study is the first to report the presence of TCRαβ^+^CD8αα^+^ IEL in chickens, which constituted 20-30% of total T cell IEL. While the exact role of TCRαβ^+^CD8αα^+^ IEL in mucosal immunity is not fully understood, there is some evidence suggesting they have immune regulatory functions through the expression of TGF-β3, lymphocytes activation gene-3, and fibrinogen-like protein 2 (51). Therefore, the TCRαβ^+^CD8αα^+^ IEL protect immune compromised mice against colitis development following the adoptive transfer of CD4^+^CD45RB^hi^ T cells (52). The robust increase in the number of natural TCRαβ^+^CD8αα^+^ IEL in EM/CP groups at 1 dpi and their potential regulatory function, might explain relatively low lesion scores and mild signs of NE disease.

Another significant alteration in natural IEL was the increase in the number of TCRγδ^+^ cells following *C. perfringens* infection at 1 dpi (**Figure 5**). Previous research has reported a high frequency of TCRγδ+ cells in the cecal tonsils following infection with a virulent-*C. perfringens* strain, suggesting these cells play a crucial role in mediating anti-*C. perfringens* immunity in chickens (53). Furthermore, ex-vivo stimulation of the small intestinal TCRγδ^+^ cells with *C. perfringens* increased the frequency of these cells (54). In the current study, the majority of jejunal TCRγδ^+^ IEL are expressing either CD8αα homodimers or CDαβ heterodimers, identifying two-distinct subsets of IEL (40). In contrast to mice and humans, TCRγδ^+^CD8αβ^+^ cells are particularly interesting because they are unique to the chicken IEL compartment, and their role in mucosal immunity is yet to be elucidated. Our data indicated an induction of TCRγδ^+^CD8αα^+^ IEL in EM/CP groups compared to all other groups, and the number of TCRγδ^+^CD8αβ^+^ IEL increased in the EM/CP group compared to the EM group at 1 dpi. The main function of TCRγδ^+^ IEL is to protect the intestinal epithelium by inhibiting an early invasion of resident and pathogenic microorganisms and maintaining intestinal homeostasis by limiting excessive inflammation and tissue damage during infection (55, 56). TCRγδ^+^ IEL produce a variety of effector molecules such as pro-inflammatory cytokines (IFN-γ and TNF-α), anti-inflammatory cytokines (TGF-β and IL-10), wound healing factors (TGF-β, prothymosin β4, and keratinocyte growth factor), cytotoxic enzymes (granzymes), and antimicrobial peptides (11). In *Salmonella*-infected chickens, both TCRγδ^+^CD8αα^+^ and TCRγδ^+^CD8αβ^+^ cells express IFN-γ mRNA, suggesting these cells can promote the phagocytic capability of macrophage to clear infection (57). Therefore, the upregulation of INF-γ expression (**Figure 8**) and augmentation of TCRγδ^+^ subsets in the jejunum of EM/CP group imply the essential role of TCRγδ^+^ IEL in enhancing the bactericidal function of macrophages at the early infection stage.

The third cell group of natural IEL that experienced a significant increase following *C. perfringens* infection at 1 dpi, was TCR^neg^ cells (**Figure 6**). These cells belong to the innate immune cells and are not fully characterized in chickens. Previous studies have identified NK cells as a distinct TCR^neg^ IEL population in chickens during embryonic and early life (20, 21). In mice and humans, several subsets of TCR^neg^ IELs have been identified and characterized with vital functions in the mucosal immunity (8). TCR^neg^ IEL are composed of innate lymphoid cells expressing NK receptors (NKP46, NK1.1, and NKP44) (15–17), and lymphocytes expressing intracellular CD3γ. The later lymphocytes are further divided according to the expression of CDαα into intracellular CD3 (CD8α^−^) and iCD8α (CD8α^+^) (18, 19). The iCD8α cells produce a diverse array of effector molecules, such as monocyte chemotactic protein 1 (MCP-1), IFN-γ, and OPN, and possess antigen processing and presentation, cytotoxicity, and phagocytosis, highlighting their crucial role during early immune responses (19). Our results indicate, for the first time, that chickens harbor iCD8α cells in the intestine and their number increased following *C. perfringens* infection (**Figure 6**), suggesting that these cells might confer protection against *C. perfringens*, similar to their protective role against *Citrobacter rodentium* infection in mice (19).

Our previous results in mice indicated that iCD8α cells through OPN mediate the homeostasis of IEL subpopulations by promoting their survival in a CD44-dependent manner, proliferation, and migration to intestinal epithelium (31). For example, OPN-knockout mice exhibit a reduction in the numbers of TCRαβ^+^CD4^+^, TCRαβ^+^CD4^+^CD8αα^+^, TCRαβ^+^CD8αβ^+^, TCRαβ^+^CD8αα^+^, and TCRγδ^+^ IEL in the small intestine and colon compared to wild-type mice. Moreover, OPN sustains proper expression of Foxp3 on regulatory T cells in the intestine and therefore, protect against colitis (31). In the present study, induction of subclinical NE was associated with upregulation of OPN mRNA level and high number of iCD8α IEL in the jejunum (**Figure 8**). These findings underscore the importance of iCD8a cells and OPN in intestinal health and warrant further investigation of their roles during mucosal immune responses.

In summary, in this report, we provide evidence supporting the immunological significance of natural IEL subsets, specifically TCRγδ^+^, TCRαβ^+^CD8αα^+^, and TCR^neg^, including iCD8a cells, in the early immune response of chickens to NE. Furthermore, we report, for the first time, that chickens harbor fractions of TCRαβ^+^CD8αα^+^ and iCD8a cells in IEL compartments, suggesting their potential role in conferring protection against *C. perfringens* infection. However, the precise functions and mechanisms of these IEL populations in the context of intestinal inflammation in chickens warrant further investigation.

## Data availability statement

The raw data supporting the conclusions of this article will be made available by the authors, without undue reservation.

## Ethics statement

The animal study was approved by The Ohio State University’s Institutional Animal Care and Use Committee. The study was conducted in accordance with the local legislation and institutional requirements.

## Author contributions

SM: Data curation, Investigation, Methodology, Writing – original draft. SH: Data curation, Investigation, Methodology, Resources, Writing – review & editing. BS: Data curation, Investigation, Methodology, Writing – review & editing. LB: Methodology, Writing – review & editing. AN: Conceptualization, Formal Analysis, Funding acquisition, Investigation, Methodology, Project administration, Resources, Software, Supervision, Validation, Visualization, Writing – review & editing.
