# Supplementary figures and images for: Natural intraepithelial lymphocyte populations rise during necrotic enteritis in chickens
Source: Front Immunol. 2024 Feb 22;15:1354701. doi: 10.3389/fimmu.2024.1354701 (PMC10917894; doi:10.3389/fimmu.2024.1354701)

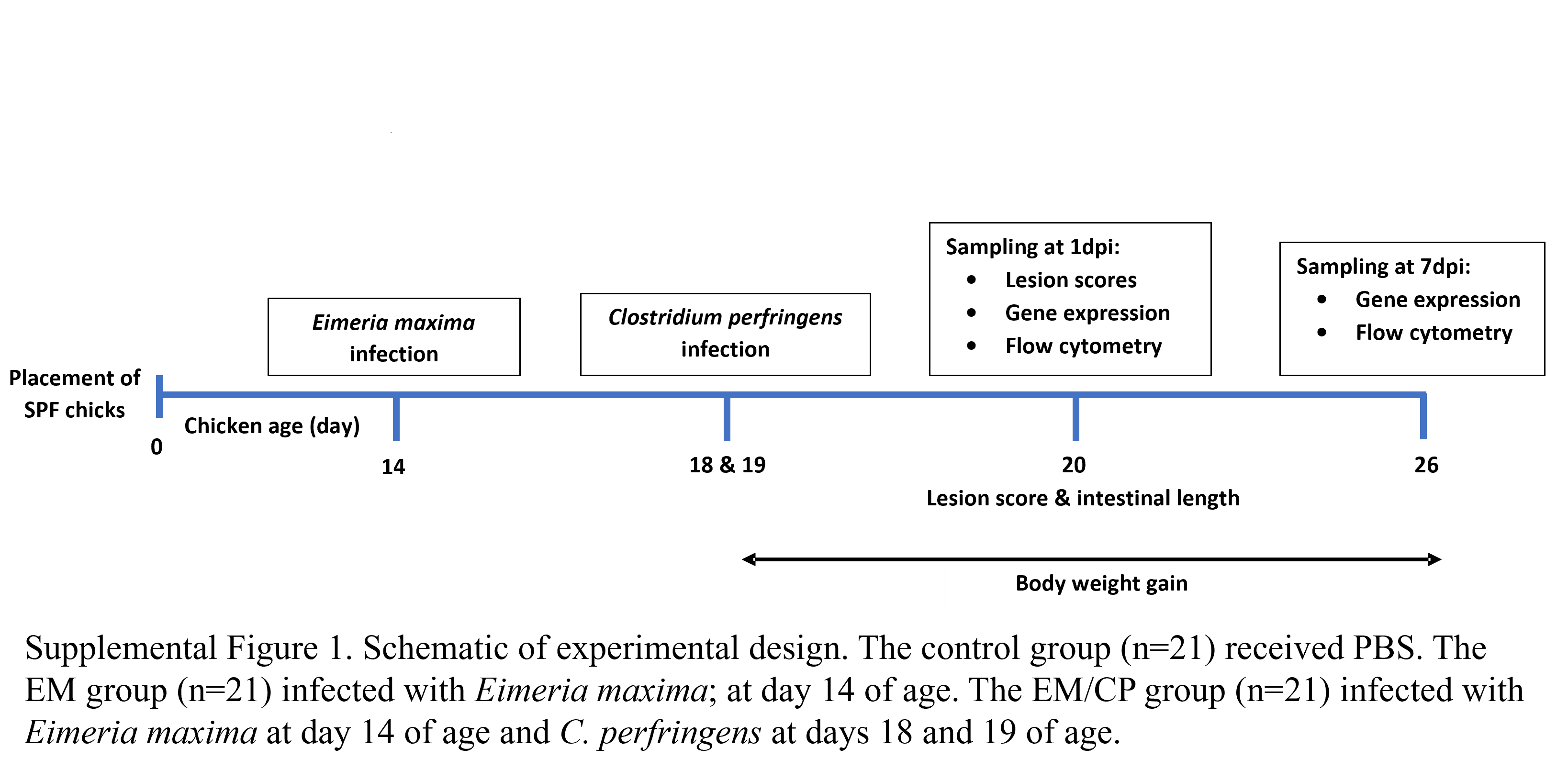

Supplement: Supplementary file 1 [file Image_1.tif]
